# Microbiota and Alzheimer’s disease: mechanistic insights from a multi-organ perspective

**DOI:** 10.1186/s40035-026-00541-9

**Published:** 2026-02-11

**Authors:** Jiewen Liao, Hanmeng Mou, Shilin Luo, Lu Shen, Bin Jiao

**Affiliations:** 1 Department of Neurology, Xiangya Hospital, Central South University, Changsha, 410008 China; 2 National Clinical Research Center for Geriatric Disease (Xiangya Hospital), Central South University, Changsha, 410008 China; 3 Engineering Research Center of Hunan Province in Cognitive Impairment Disorders, Central South University, Changsha, 410008 China; 4 Hunan International Scientific and Technological Cooperation Base of Neurodegenerative and Neurogenetic Diseases, Xiangya Hospital, Central South University, Changsha, 410008 China; 5 Brain Research Center, Central South University, Changsha, 410008 China; 6 FuRong Laboratory, Changsha, 410078 China

**Keywords:** Gut microbiota, Microorganisms, Alzheimer's disease, Diagnosis, Microbiota interventions, Microbiome-based therapeutics

## Abstract

Alzheimer’s disease (AD) is a progressive neurodegenerative disorder driven by multifactorial mechanisms. Increasing evidence suggests that dysbiosis, a term denoting an imbalance in the composition of the microbiota, may play a pivotal role in the pathogenesis of AD across multiple bodily sites, including the gut, oral cavity, nasal passages, lungs, and skin. Microbial imbalances may promote neuroinflammation, immune dysfunction, and metabolic disturbances through complex host–microbiota networks. This review synthesizes current advances in the understanding of microbiota-driven modulation of AD, introduces the “Multi-Axis Co-Regulation” concept, and evaluates microbial biomarkers for early diagnosis. Finally, the translational potential of microbiota-targeting interventions, including probiotics, dietary modulation, fecal microbiota transplantation, and oral microbiome-based therapies, are discussed, which represent novel strategies for precision prevention and treatment of AD.

## Introduction

AD is a progressive neurodegenerative disease, ranging from modest early-onset spontaneous cognitive abnormalities to severe late-onset neurological and psychiatric symptoms such as executive function, complicated attention, and language dysfunction [1, 2]. It is one of the major causes of dementia and death in the world [3]. According to the World Health Organization, about 55.2 million individuals worldwide suffered from dementia in 2022. The prevalence among individuals aged 60 and above varies by region, with Southeast Asia reporting a prevalence of 2.9%, Europe at 6.5%, and other regions ranging between 3.1% and 5.7%. It is predicted that there will be 78 million dementia sufferers by 2030 [4].

AD is pathologically characterized by β-amyloid (Aβ) deposition and abnormal tau protein phosphorylation. Besides the two key players, microbiota dysbiosis is also believed to be involved in the onset and progression of AD [5]. The human body is not a sterile vessel; microbial communities inhabit at numerous sites, particularly within the gut, oral cavity and lungs. These communities play a pivotal role in maintaining the systemic homeostasis, and their dysregulation is closely associated with a variety of diseases, including neurodegenerative disease [5, 6]. Extending from previous reviews that have focused on these microbial sites in isolation, this review synthesizes emerging evidence to propose a novel, integrative framework: the "Multi-Axis Co-Regulation" of the gut, oral, and lung microbiomes. This perspective is crucial for understanding their collective impact on AD pathogenesis and for guiding the development of comprehensive diagnostic and therapeutic strategies.

The gut microbiota is the largest microbial population in the human body, consisting of bacteria, fungi, and viruses. The majority of microorganisms live in the ileum and colon [7]. The intestinal flora has a significant impact on human health and disease. In addition to regulating intestinal function, it directly or indirectly affects the communication between the gastrointestinal tract and the central nervous system (CNS) in both healthy and diseased states by acting on the immune system, metabolism, and epigenetic inheritance of the host. In addition to bacteria, fungi and viruses seem also associated with AD [8, 9].

The oral cavity, including the tongue, dental plaque, cheeks, gums, and oral mucosa, contains more than 700 types of bacteria [10, 11]. Apart from the gastrointestinal microbiome, oral flora is thought to be the most diverse in the human body. These florae can interact with the host or with each other, enter the bloodstream through gingival inflammation or damage from brushing, and impact the central nervous system [12–15].

Microorganisms are also present in the human respiratory system. Recent research has challenged the long-held belief that the typical lung is a sterile organ [16]. Lung microbiota are comprised of bacteria, fungi, and viruses [17, 18]. The fungi have relatively low biomass and diversity in the lungs [19]. Eukaryotic viruses have also been found in the lungs of healthy individuals. These viruses are involved in the regulation of both health and disease, and respiratory symptoms may arise when the viral load reaches a particular threshold [20].

Notably, the alterations in gut microbiota exhibit distinct patterns across different neurodegenerative diseases. For instance, in Parkinson's disease (PD), consistent findings include reduced abundance of *Bacteroidetes* and *Prevotellaceae*, accompanied by an increase in *Enterobacteriaceae* and *Bifidobacterium* [21, 22]. In contrast, mouse models of Huntington's disease (HD) not only display changes similar to those observed in AD—such as increased abundance of *Bacteroidetes* and decreased proportion of *Firmicutes* [23, 24]—but also exhibit gut fungal dysbiosis and altered fungal-bacterial interactions [25].

**Fig. 1:**
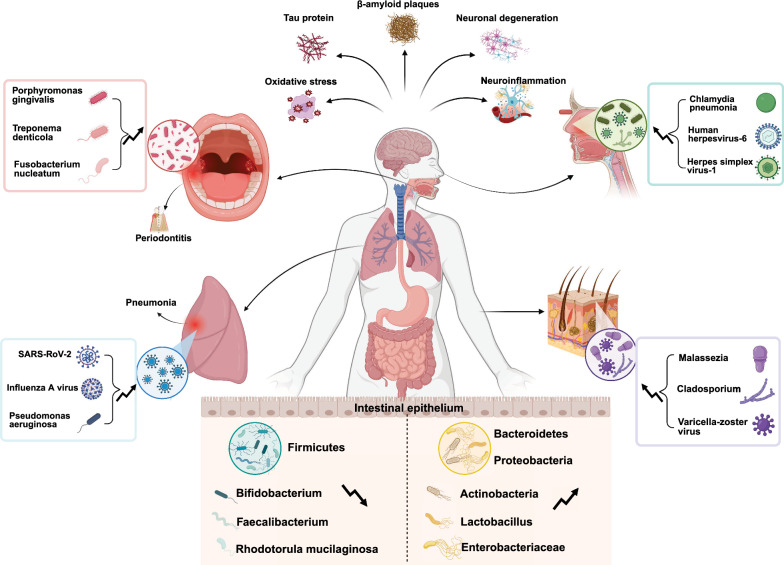
Microbial changes in Alzheimer's disease. Microbial dysbiosis associated with AD involves notable changes in the composition and diversity of microbiota in the oral cavity, lungs, gut, skin, and nose

Current research predominantly centers on the gut-brain axis; however, significant gaps remain in understanding the synergistic and cumulative effects of microbial communities across multiple sites—such as in the oral cavity and lungs—on AD pathogenesis. In this review, we systematically synthesize alterations in the microbiome across different ecological niches in AD patients (Table 1; Fig. 1) and further elucidate the specific mechanisms by which microbial communities from various body sites influence AD. Additionally, we explore novel diagnostic approaches based on the microbiome–AD relationship and the translational prospects of microbiome-targeted therapeutic strategies, thereby offering a theoretical foundation for clinical translation.

**Table 1:** Comparison of microbiome profiles between Alzheimer’s disease patients and healthy adults

Organ	Microbiota in healthy adult	Microbiota in AD
Gut	*Firmicutes* and *Bacteroidetes* making up about 95% of the entire microbiota. *Proteobacteria*, *Actinobacteria*, *Clostridium*, and *Spirochaetes* are next in line [331, 332]	*Firmicutes* decreased *Bacteroidetes*, *Actinobacteria*, and *Proteobacteria* increased *Lactobacillaceae* in the *Firmicutes* phylum and *Bifidobacterium* in the *Actinobacteria* phylum decreased [333–336]
Oral	*Actinobacteria, Bacteroidetes, Firmicutes, Fusobacteria, Pseudomonas, Saccharibacteria, and Spirochaetota* [337, 338]	*Firmicutes, Fusobacteria*, and *Spirochaetes* increased *Bacteroidetes, Actinobacteria*, and *Proteobacteria* generally decreased *Prevotella* and Porphyromonas of the *Bacteroidetes* phylum and *Moraxella* of the *Proteobacteria* phylum increased [339–341]
Lung	Bacteria: *Pseudomonas, Streptococcu*s, *Prevotella, Fusobacterium, Haemophilus, Veillonella, Porphyromonas* [342, 343] Fungi: *Ascomycetes* and *Streptomyces* [344] Eukaryotic viruses: human bocavirus, rhinovirus, and WU polyomavirus [345]	*Chlamydia pneumoniae* infection increases the risk of AD by more than five times [346] *Bordetella pertussis* infection in mice is linked to Aβ deposition Respiratory Klebsiella pneumoniae infections are also linked to AD [347]
Nose	Bacteria: *Actinobacteria, Firmicutes, Proteobacteria* Fungi: *Exobasidiomycetes, Malassezia, Aspergillus, Malassezia restricta, Malassezia pachydermatis, Malassezia globosa*, *Schizophyllum* and *Cryptococcus* Virus: human influenza A virus (IAVs), respiratory syncytial virus (RSV), COVID-19, herpes simplex virus (HSV), adenovirus, Murid herpesvirus-4, varicella zoster virus [348, 349]	*Chlamydia pneumonia* is present in some AD olfactory bulbs [349] Human herpesvirus-6 (HHV-6) levels are elevated in the brains of AD patients [348]
Skin	*Actionobacteria, Bacteroidetes*, *Firmicutes*, and *Proteobacteria* [350]	Fungal skin infections caused by *Cladosporium* spp. and *Malassezia* spp. may lead to the progression of AD [351, 352]

Aβ, Amyloid-beta; AD, Alzheimer's disease; COVID-19, Coronavirus disease 2019; HHV-6, Human herpesvirus-6; HSV, Herpes simplex virus; IAVs, Influenza A viruses; RSV, Respiratory syncytial virus

## Multifaceted interactions of the gut-brain axis in AD

The Microbiota–Gut–Brain Axis consists of gut microbes, the nervous system including the central nervous system, the neuroendocrine system (e.g., hypothalamic–pituitary–adrenal axis), the neuroimmune system, the autonomic sympathetic and parasympathetic nervous systems, and the immune system.

Bidirectional signaling exists between the gut and the brain. Descending signaling primarily occurs through two pathways of the autonomic nervous system: the hypothalamic–pituitary–adrenal (HPA) axis coupled with the sympathetic-adrenal axis (which regulates gut-associated lymphoid tissue), and the descending monoaminergic pathway (which modulates spinal reflex gain and dorsal horn excitability). Ascending gut-to-brain signaling encompasses neural transmission via the enteric nervous system, endocrine and paracrine signals, the sympathetic-adrenal axis, the descending monoaminergic pathway, and immune-mediated signals [26].

**Fig. 2:**
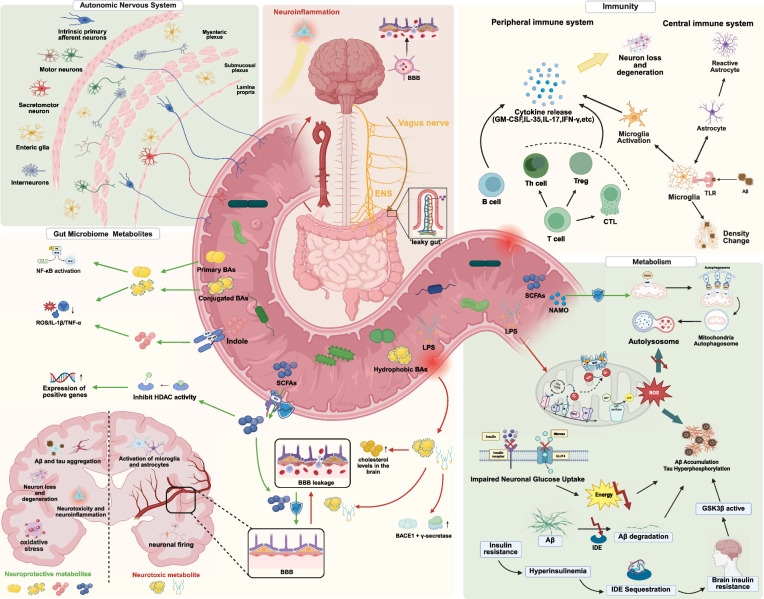
Involvement of gut microbiota in Alzheimer’s disease pathogenesis. The gut microbiota plays a critical role in the development and progression of AD through multiple interconnected pathways. These include changes in systemic metabolism, microbial metabolite production, host immunity modulation, and autonomic nervous system regulation

The gut microbiota affects the development and course of AD through a variety of mechanisms, such as immune response modulation and metabolite production. These effects can be bidirectional—potentially contributing to disease progression or exerting protective effects under certain conditions (Fig. 2).

### Gut microbiome and immunity

Under physiological conditions, the commensal microbiota maintains a state of equilibrium with the host immune system. Intestinal dysbiosis can compromise the integrity of the gut barrier, facilitating the translocation of microbes and their metabolites, which subsequently engage immune pathways implicated in the pathogenesis of AD. As a result, pro-inflammatory cytokines are released, activating immune cells to maintain normal cognitive function [27–29]. We will examine how abnormal immune activity may contribute to or exacerbate the pathology of AD from both central and peripheral perspectives.

#### CNS immunity

In the CNS, microglia and astrocytes serve as the primary mediators of immune function. As innate immune cells, they play a critical role in maintaining neuronal homeostasis [30, 31]. In the following section, we will elucidate the mechanisms by which dysfunctional astrocytes and microglia contribute to the pathology of AD.

Microglia in the human brain may have two phenotypes, quiescent (M0) and activated (M1 and M2), depending on the inflammatory environment they are exposed to. There are also two different subtypes of reactive astrocytes induced by different stimuli: ischemia causes the protective A2 phenotype, which aids in CNS recovery and repair, while neuroinflammatory stimuli encourage the neurotoxic A1 phenotype [32, 33].

The number of microglia and their gene expression are influenced by the gut flora. Transplanting microbiota from young mice in old mice reduced the increased number of Iba-1^+^ microglia in the CNS of old mice [34]. Mice infected with *Helicobacter suis* showed a significant increase of Iba-1^+^ microglia in the hippocampus and displayed a loss of short-term memory [35]. Another animal study showed that administering *Bacteroides fragilis* in APP/PS1-21 mice lowers the expression of *Trem2* in microglia, a gene important for phagocytic clearance of plaques by microglia [36]. In C/EBPβ transgenic mice transplanted with gut microbiota from AD patients, an increase in *Bacteroides Fragilis* in the gut flora was found to trigger the release of pro-inflammatory cytokines from microglia [37]. Gut flora can influence astrocytes through both microglia-dependent pathways and microglia-independent mechanisms. Antibiotics-mediated gut microbiome perturbation leads to reduced astrocytosis as indicated by glial fibrillary acidic protein staining, which is independent of microglia, while the antibiotics-mediated astrocyte morphological changes are related to microglia [38]. Additionally, gut microbes can influence astrocytes by altering cytokines. A study using CRISPR-Cas9-based genetic perturbations in mice, showed that gut microbiota regulates IFN-γ expression in meningeal NK cells, thereby acting on specific astrocyte subpopulations to reduce inflammation in the CNS [39].

One of the characteristics of neuroinflammation in AD is the accumulation of activated microglia in brain lesion sites. The activated microglia have a neuroprotective effect in the early stages of the disease by removing excessive Aβ deposits. In the later stages, however, the overactivated microglia cause neuronal damage by overproduction of proinflammatory cytokines (IL-1, TNF-α, etc.) [40]. Additionally, in P301S-tau mice, microglia selectively phagocytose neurons harboring tau aggregates, a process that simultaneously promotes neuronal death and facilitates tau propagation [41, 42]. Like microglia, astrocytes directly or indirectly participate in the clearance and degradation of Aβ plaques through proteolysis or autophagy [43–46]. Reactive astrocytes significantly proliferate in AD mouse models and human patients and gather around Aβ plaques [47–49].

An essential part of the mammalian innate immune system is the toll-like receptors (TLRs). TLRs are expressed mainly in microglia within the CNS [50, 51]. TLRs may serve as the initial triggers of neuroinflammation in the CNS [52–54]. Lipopolysaccharides (LPS) activate TLR4 in microglia after crossing the blood–brain barrier (BBB). Overactivation of TLR4 subsequently triggers the downstream NF-κB signaling pathway, leading to the release of pro-inflammatory cytokines that worsen neuroinflammation. Animal studies have shown that fecal microbiota transplantation (FMT) can decrease TLR4 expression in both the gut and the brain, thereby reducing microglial activation in the brain [55].

In conclusion, the dual-edged effects of microglia and astrocytes in AD complicate the therapeutic approach targeting glial cells. Thus, it is necessary to investigate the causes of the different phenotypes of glial cells at various stages of the disease. Regulation of the intestinal flora may be an effective strategy for the treatment of AD by intervening in the phenotypes of glial cells.

#### Peripheral immunity

T cells and B cells, two types of adaptive immune cells, are primary components of the peripheral immune system. Changes in these cells and the crosstalk between them are critical in the development of AD neuropathology [56].

In the inflammatory state, the activated T cells in the brain release cytokines and set off an effector immunological response, which increases the permeability of the BBB. After that, a significant number of immune cells enter the brain, exacerbating neuroinflammation [57–59].

The gut is the largest peripheral lymphoid organ in humans [60]. Gut flora can affect intestinal immune cell differentiation and function. *Firmicutes* and *Bacteroides* lead to a decrease of CD3^+^ T, CD4^+^ T, and CD8^+^ T cells and an increase of regulatory T cells (Tregs) [61]. *Klebsiella* spp. are strong inducers of Th1 cells when they colonize in the intestinal tract [62]. Additionally, *Lactobacillus plantarum* strain 06CC2 enhances the immune response of Th1 cells through intestinal immunization [63].

Gut flora can influence microglia status through affecting the adaptive immune cells. For instance, administering *Bifidobacterium fragilis* in APP/PS1-21 mice prevented CD4⁺ T cell production of GM-CSF (granulocyte–macrophage colony-stimulating factor) that can stimulate microglial proliferation [36]. During AD progression, alterations in the composition of gut flora lead to the accumulation of phenylalanine and isoleucine in the periphery. In AD mouse models, this promotes the growth and differentiation of proinflammatory Th1 cells and enables their local interaction with M1 microglia, which in turn drives microglial differentiation toward a proinflammatory state [64].

There is growing evidence that T cell subsets play a complex role in the pathophysiology of AD, with varying functional outcomes. In a mouse model of AD, Th1 cells were demonstrated to stimulate microglial activation and Aβ deposition [65]. The role of Tregs is still controversial. While their expansion restores cognitive function and their depletion accelerates cognitive deficits in APP/PS1 and 3 × Tg-AD models [66–69], in 5 × FAD mice, their depletion improves Aβ plaque clearance and ameliorates pathology [70]. Another pathogenic feature of AD is CD8^+^ T cell infiltration and concentration in afflicted brain areas, along with enhanced release of proinflammatory cytokines in both AD patients and animal models of tauopathy [71, 72]. In AD mouse models, Th cells activate microglia by secreting cytokines, including IFN-γ, IL-6, and IL-17, while infiltrating CD8⁺ T cells further enhance microglial activation through the release of granzyme and perforin. Collectively, these immune cells increase the production of proinflammatory mediators such as IL-1β and IFN-γ [65, 67, 73, 74].

Antigens derived from the gut microbiota can promote B-cell activation and differentiation via two distinct pathways: they can directly stimulate B cells through BCR- or TLR-mediated signaling, leading to plasma cell differentiation, or indirectly modulate B-cell responses by acting on T cells, dendritic cells, and other immune cells, which subsequently influence B-cell activation and differentiation [75]. The involvement of B lymphocytes in the pathophysiology of AD is still poorly understood, and current research has provided conflicting results. According to new research, B cells may have harmful impacts on AD. Mature B cells were found in the brains of AD mice using immunophenotyping [76].

In conclusion, immune cells function as biological sensors, translating intestine signals into neural regulatory signals. We propose the "Microbiota-Immune-Brain Axis", emphasizing the bridging role of the immune system in the pathogenesis of AD. Building on the mechanistic understanding of the gut-immune axis, a key translational pathway lies in designing integrated interventions that simultaneously modulate host immunity and microbial ecology. This encompasses the rational design of combination therapies and the pioneering of microbiota-centric approaches like next-generation vaccines.

### Gut microbiome-derived metabolites

Recent studies have shown that gut microbial metabolites play a crucial role in the onset and progression of AD. Gut microbiota can produce various bioactive molecules through metabolism, including LPS, short-chain fatty acids (SCFAs), bile acids (BAs), and indole derivatives.

#### LPS

LPS are major metabolites and prototypical endotoxins produced by gut-resident Gram-negative bacteria such as *Desulfovibrio* and *Escherichia*. These molecules play a central role in mediating gut-brain communication and have been increasingly associated with the development of AD.

A number of cohort studies have found that patients with AD have higher blood and tissue levels of LPS than the general population, and the blood levels of LPS show a trend of increase as people age [77–81]. Serum levels of endotoxins have been reported to correlate with the levels of Aβ_1-42_ and tau in the CSF, and with memory impairment in patients with MCI and AD [79]. In addition, endotoxins are far more prevalent in the brains of AD patients at autopsy, primarily accumulating in the hippocampus. In some late-stage AD patients, the levels of endotoxins can be up to 26 times higher compared to age-matched individuals without dementia [82]. This accumulation may lead to memory impairment via microglial TLR4-driven neuroinflammation and synaptic dysfunction [83]. Memory impairment mediated by hippocampal vulnerability to immunity is also evident in neuroimmune disorders, such as the isolated retrograde amnesia in CASPR2 encephalitis [84] and the chronic cognitive deficits following immune checkpoint inhibitor-induced limbic encephalitis [85]. However, in AD, this hippocampal perturbation stems primarily from chronic innate immune activation due to peripheral endotoxin exposure, rather than adaptive autoimmunity.

LPS may be involved in the pathogenesis of AD in multiple ways. Aβ protein level and APP expression in the brain are increased in mice receiving intraperitoneal LPS injection [86, 87]. LPS appears to promote amyloid pathology at the level of Aβ synthesis, Aβ aggregation, and Aβ neurotoxicity. Possible mechanisms include that LPS increases APP expression and increases β- and γ-secretase activity [88, 89]. LPS can disrupt the BBB, leading to decreased efficiency of Aβ clearance and possibly triggering a decrease in efflux of Aβ, resulting in Aβ accumulation [90]. At the same time, Aβ itself has antimicrobial properties. LPS-mediated intracranial infection is likely to stimulate the production and aggregation of Aβ, which in turn prevents the infection [91, 92]. Other studies showed that Aβ pathology exacerbates brain cytokine responses following LPS administration in AD mice and that Aβ42 peptide facilitates LPS entry into human neurons [93, 94]. The potential synergistic effects of Aβ and LPS may include: induction of neuroinflammation; producing neurotoxicity to disrupt gene expression; activation of microglia with impairment of their ability to clear amyloid plaques; and triggering synaptic dysfunction, neuronal apoptosis, and exacerbation of cognitive deficits [94–98].

LPS may further accelerate tau transmission by compromising the BBB integrity [99]. In addition, LPS triggers synaptic, neuronal, and cognitive impairments through microglia-mediated pathways, including: (1) synaptic pruning via microglial phagocytosis, (2) direct neuronal engulfment, (3) production of neurotoxic reactive oxygen/nitrogen species, and (4) induction of neuronal tau hyperphosphorylation and aggregation [97, 100–110].

There are interactions between genetic or non-genetic factors of AD and LPS. The *APOE4* variant is one of the risk factors for AD. LPS strongly induces the expression of ApoE in mice, which then binds to LPS and redirects it from macrophages to hepatocytes, thereby removing LPS from the blood [111, 112]. Individuals carrying the *APOE4* variant are more sensitive to injected LPS compared to those carrying *APOE3* [113, 114]. Other AD pathogenic genes also have direct or indirect connections with LPS.

With deepening of research on the relationship between LPS and AD, future studies examining how LPS, the brain microbiome, and the BBB function interact may provide new perspectives for developing novel therapeutic strategies.

#### SCFAs

SCFAs are generated from the microbiota and are mostly found in the human colon [115]. They are produced by anaerobic microorganisms that ferment indigestible carbohydrates, with the remainder from dietary intake and metabolism (such as protein metabolism). Subsets of anaerobic bacteria, particularly members of the genera *Clostridium*, *Eubacterium*, and *Butyrivibrio*, produce high levels of SCFA in the gut [116]. APP/PS1 transgenic mice have lower levels of butyric and isobutyric acids in their brains and feces [117]. Some studies have shown that serum levels of SCFAs are similarly reduced in patients with AD [118, 119]. Notably, the reduction of SCFAs is not unique to AD; similar decreases have been observed in PD, which may contribute to intestinal inflammation, impaired barrier function, and constipation in PD. In AD, however, SCFAs appear to play a more disease-specific role by modulating tau acetylation and microglial function. Interestingly, a study using a presymptomatic mouse model of HD reported upregulation of the intestinal butyrate metabolic pathway, suggesting increased production of butyrate, although the circulating butyrate level remained unchanged. This indicates that early disorder of the intestinal environment in neurodegenerative diseases may lead to compensatory or disordered activation of the butyrate metabolic pathway in microbiota [120].

SCFA, indole derivatives, and bile acid derivatives produced by probiotics can promote ZO-1 expression and reduce intestinal permeability. Butyrate can activate the Wnt signaling pathway in intestinal epithelial cells, allowing for their proliferate and repair while maintaining the integrity of the intestinal barrier [121–125]. Transplanting butyrate-producing bacteria into the gut of germ-free mice can reverse the alterations in BBB permeability [126]. In APP/PS1 mice, SCFAs attenuate the inflammatory response by upregulating G protein-coupled receptor 41 and downregulating the NF-κB pathway, thereby improving BBB function [127]. The above results suggest that disturbances in the intestinal flora may decrease the production of SCFAs, increasing barrier permeability, further leading to the entry of toxins (including LPS) into circulation.

SCFAs are also a key player in the Aβ and tau hypotheses. Clinical studies have shown that the Aβ levels in AD patients are positively correlated with serum acetate and valerate concentrations and negatively correlated with butyrate levels [128]. SCFAs can inhibit the aggregation of Aβ by suppressing APP protein expression, reducing reactive oxygen species (ROS) through nuclear factor erythroid 2-related factor 2 (Nrf2) stabilization, and lowering cholesterol levels [129–131]. SCFAs exhibit a protective effect against the neurotoxicity caused by the aggregation and accumulation of Aβ. Sodium butyrate, in particular, promotes mitochondrial function and cell proliferation, thereby ameliorating Aβ-induced damage in N2a cells [131]. Sodium propionate exerts a protective effect against Aβ-induced neurotoxicity by inhibiting the production of inducible nitric oxide synthase and cyclooxygenase-2 (COX-2) [132]. Studies related to tau have also explored the effects of SCFAs on tau phosphorylation, but the conclusions are inconsistent and contradictory [133, 134].

Dysfunction of the HPA axis has been associated with cognitive decline, aging, immunological failure, and systemic inflammation [135]. Administration of SCFAs decreased the HPA‐axis reactivity in stressed mice [136]. Studies in human subjects have provided consistent results [137]. SCFAs inhibit neuroinflammation, and decreased levels of SCFAs in the gut of AD patients may lead to increased neuroinflammation and cognitive decline [138]. In vitro studies also showed that SCFAs reduce the expression of IL-1β, IL-6 and TNF-α, and increase the transcription of anti-inflammatory factors such as TGF-β1 and IL-4 [139, 140]. SCFAs may alleviate related neuroinflammation by regulating the structure and maturation of microglia, as well as reducing the secretion of IL-1β from microglia [139–142].

Some SCFAs can cross the BBB and exert protective effects against AD by inhibiting histone deacetylases (HDACs) activity [143, 144]. Alterations in HDACs are closely associated with neurodegenerative diseases, including AD [145]. Thus, it is hypothesized that SCFAs may influence the expression of relevant genes associated with cognitive impairment through inhibition of HDAC activity, thereby protecting against AD. In a mouse model of AD, sodium butyrate ameliorated cognitive impairment through increasing expression of memory consolidation genes such as *MYST4, Marcksl1, GluR1, SNAP25*, and *SHANK3* [146]. In conclusion, further studies are needed to clarify the specific mechanisms underlying the effects of SCFAs in AD, especially their effects on neuroinflammation, Aβ deposition, and neurodegenerative pathology. Modulating the gut microbiota or supplementing SCFAs is an important direction for preventing and slowing the progression of AD.

#### Bile acid

Bile acids are the main components of bile. They are eventually released into the bile as conjugated bile acids. Once secreted into the small intestine, bile acids undergo a series of biotransformation by gut microbiota to generate secondary bile acids. Most bile acids are reabsorbed in the ileum and returned to the liver via the portal venous circulation to inhibit bile acid synthesis [147–149]. The gut microbiota involved in bile acid metabolism include genus *Clostridium*, *Ruminococcaceae* family, *Firmicutes*, *Proteobacteria*, genus *Bacteroides*, *Lachnospiraceae*, and genus *Eggerthella* [150, 151].

Three primary bile acids, cholic acid (CA), ursodeoxycholic acid, and chenodeoxycholic acid, have been found to be protective against AD (Table 2). A study involving 182 healthy individuals and 136 AD patients found that the serum CA levels are significantly reduced in AD patients. The reduced serum CA significantly increases Aβ burden, which in turn causes a considerable decrease in cerebral blood flow.

**Table 2:** Gut microbiome-derived metabolites in Alzheimer’s disease pathogenesis

Metabolites			Main sources	Effect on AD	Mechanism
Lipopolysaccharides			Produced by *Desulfovibrio* and *Escherichia* in the intestinal tract	Adverse factors	Enhances Aβ production/aggregation & tau pathology[353, 354] Disrupts BBB, impairing clearance [355] Synergizes with Aβ to induce neuroinflammation and synaptic/neuronal damage [95, 356–359] Activates microglial phagocytosis, neurotoxin release, and tau hyperphosphorylation [358] Modulated by AD risk genes (e.g., *APOE4*) [360]
Short-chain fatty acids			Produced by gut anaerobic microorganisms fermenting indigestible carbohydrates, with the remainder derived from dietary intake and metabolism (e.g., protein breakdown)	Protective factor	Barrier Protection: Enhance gut (ZO-1) and BBB integrity; butyrate-producing bacteria reverse BBB permeability [361] Anti-inflammatory: Upregulate GPR41, downregulate NF-κB [362]; reduce IL-1β, IL-6, TNF-α; modulate microglial function [363, 364] Aβ Modulation: Inhibit Aβ aggregation via APP suppression, Nrf2-mediated ROS reduction, and cholesterol lowering; butyrate/propionate counteract Aβ neurotoxicity [365, 366] Epigenetic Regulation: Cross BBB, inhibit HDACs, upregulate memory-related genes (e.g., MYST4, GluR1) [367] HPA Axis Regulation: Suppress stress-induced HPA-axis hyperactivity [368]
Bile acids	Primary bile acids	CA	Direct synthesis from cholesterol in hepatocytes	Protective factor	Decreasing γ-secretase activity, increasing Aβ clearance factors [369]
		UDCA			Reducing apoptosis, ROS, TNF-α, lactate dehydrogenase, IL-1β, and cytochrome c peroxidase production [370]
		CDCA			Enhancing insulin signaling to reduce neurotoxicity and cognitive decline [371, 372]
		TUDCA	UDCA conjugates with taurine, and is released into bile as conjugated bile acids		Reducing Aβ deposition Inhibiting glycogen synthase kinase-3β (GSK-3β) activity and decreasing tau phosphorylation Decreasing ROS and IL-1β production and suppressing neuroinflammation Inhibiting mitochondrial disturbance [373, 374]
	Secondary bile acids	DCA	Primary bile acids secreted into the small intestine undergo a series of biotransformation reactions by the gut microbiota to generate secondary bile acids	Adverse factors	Disrupting the BBB by activating Rac1 through phosphorylation [375, 376] When a certain concentration is reached, the FXR is activated, leading to increased cholesterol levels in the brain and promoting the accumulation of Aβ [377, 378] Upregulating APP production by acting on TGR5 receptor [379]
		LCA			When a certain concentration is reached, the FXR is activated, leading to increased cholesterol levels in the brain and promoting the accumulation of Aβ [377, 378]
Indoles			Over 85 species of Gram-positive and Gram-negative bacteria (e.g., *Escherichia coli* and *Bacteroides*) produce indole through tryptophan hydrolysis via tryptophanase	Protective factor	Aβ & Tau Modulation: IAA enhances microglial/astrocytic phagocytosis of Aβ and ameliorates tau hyperphosphorylation, improving cognitive function [380, 381] Anti‑inflammatory: IAA upregulates AHR in glial cells, inhibits NF‑κB and NLRP3 inflammasome activation, and reduces pro‑inflammatory cytokines (TNF‑α, IL‑6, IL‑1β, IL‑18) [380, 382] Antioxidant: IAA activates Nrf2 pathway and modulates DR3/IKK/NF‑κB signaling to alleviate oxidative stress and neuroinflammation [383]

Aβ, Amyloid-beta; AD, Alzheimer's Disease; AHR, Aryl Hydrocarbon Receptor; APOE4, Apolipoprotein E4; APP, Amyloid Precursor Protein; BBB, Blood–Brain Barrier; CA, Cholic Acid; CDCA, Chenodeoxycholic Acid; DCA, Deoxycholic Acid; DR3, Death Receptor 3; FXR, Farnesoid X Receptor; GPR41, G Protein-Coupled Receptor 41; GSK-3β, Glycogen Synthase Kinase-3β; HDACs, Histone Deacetylases; HPA, Hypothalamic–Pituitary–Adrenal; IAA, Indole-3-Acetic Acid; IKK, IκB Kinase; IL, Interleukin; LCA, Lithocholic Acid; LPS, Lipopolysaccharides; MYST4, Iysine Acetyltransferase 6B; NF-κB, Nuclear Factor Kappa-Light-Chain-Enhancer of Activated B Cells; NLRP3, NLR Family Pyrin Domain Containing 3; Nrf2, Nuclear Factor, Erythroid Derived 2, Like 2; ROS, Reactive Oxygen Species; SCFAs, Short-Chain Fatty Acids; TGR5, Takeda G Protein-Coupled Receptor 5; TNF-α, Tumor Necrosis Factor Alpha; TUDCA, Tauroursodeoxycholic Acid; UDCA, Ursodeoxycholic Acid; ZO-1, Tight Junction Protein 1

Taurocholic acid (TCA) and tauroursodeoxycholic acid (TUDCA) are conjugated acids, which show protective effects against AD (Table 2). TCA levels in AD patients are significantly lower than those in age-matched controls [152–154]. Six months of dietary administration of TUDCA reduced Aβ aggregation and deposition in the hippocampus and prefrontal cortex, enhanced memory retention, and alleviated deficits in spatial, recognition, and contextual memory in the mouse model of acute neuroinflammation [155, 156].

Deoxycholic acid (DCA) and lithocholic acid (LCA) are secondary BAs. They may exert negative effects during the onset and progression of AD. Compared to healthy controls, patients with amnestic MCI and AD exhibit increased levels of DCA, which are associated with cognitive symptoms [157]. The levels of LCA in AD patients are also significantly higher than those in healthy controls [158]. As serum bile acid levels increase, brain bile acid concentrations also rise correspondingly, as brain bile acids are primarily derived from the periphery, which enter the brain via passive diffusion, particularly for more lipophilic species [159, 160]. After entering neurons, bile acids act on various nuclear receptors, including the farnesoid X receptor (FXR) [161]. Specifically, they can inhibit corticotropin-releasing hormone (CRH) release through activation of hypothalamic FXR. Alternatively, bile acids may interact with the G protein-coupled bile acid receptor TGR5, which is expressed in the hypothalamic paraventricular nucleus. This interaction increases CRH involvement, thereby influencing the regulation of the HPA axis [162, 163].

Although the current experimental data support for the association between bile acids and AD, it is still unclear exactly how different bile acid types and their receptors influence the nervous system. Moreover, more studies are needed to confirm the potential of bile acid-based therapeutic strategies as a therapeutic approach for AD.

#### Indoles

Indole is a small organic molecule primarily derived from gut microbiota. It is known that over 85 species of both Gram-positive and Gram-negative bacteria, such as *Escherichia coli* and *Bacteroides*, produce indole through hydrolysis of tryptophan by tryptophanase [164, 165]. *Clostridium sporogenes* can convert tryptophan into indole-3-pyruvic acid (IPyA), which is then catalyzed by decarboxylase to produce indole-3-acetaldehyde (IAAld). IAAld is decarboxylated by members of phyla *Firmicutes, Proteobacteria, Bacteroidetes,* and *Actinobacteria* to produce indole-3-acetic acid (IAA). Additionally, IPyA can be reduced and dehydrated to generate indole-3-propionic acid (IPA) [166–168].

IAA levels in serum and CSF of APP/PS1 AD mice were found to be significantly reduced by 91.1% and 80.5%, respectively, compared to wild-type mice [169]. A study of 77 subjects comprising amnestic mild cognitive impairment, AD and healthy controls also discovered that IPyA was progressively enriched from aMCI and AD compared with healthy controls [119]. Preliminary findings suggest that the gut microbiota may manipulate tryptophan metabolites, such as indole and its derivatives, to influence the pathological accumulation of Aβ. IAA can induce activation of microglia and astrocytes to enhance Aβ phagocytosis and ameliorate hyperphosphorylated tau pathology, improving cognitive function of AD mice [169, 170]. IAA can also reduce neuroinflammation and hence have a protective effect against AD [171–175]. Further investigation revealed that IAA reduces inflammation both in vitro and in vivo by upregulating expression of AHR receptor in microglia and astrocytes, preventing activation of the NF-κB signaling pathway and formation of the NLRP3 inflammasome, and decreasing the release of inflammatory cytokines, including TNF-α, IL-6, IL-1β, and IL-18 [169, 176].

Indole metabolites, such as IAA, can alleviate oxidative stress by modulating antioxidant signaling pathways, such as Nrf2. IAA may counteract high-fat diet-induced cerebral oxidative stress and neuroinflammation by regulating the inflammatory DR3/IKK/NF-κB signaling pathway, thereby slowing the progression of AD to some extent [177].

### Gut microbiome and metabolism

Metabolic syndrome caused by disrupted gut microbiota can result in systemic chronic inflammation and weakened intestinal barriers, which can ultimately impair cognitive function [178]. Crucially, the systemic inflammation and barrier disruption converge to disrupt brain energy homeostasis, primarily by compromising mitochondrial bioenergetics and perturbing cerebral glucose utilization.

#### Mitochondrial function

Energy metabolism, neurotransmission, neuronal membrane excitability, and neuronal plasticity are all dependent on mitochondria in neurons [179–181]. Toxins produced by intestinal flora may cause mitochondrial dysfunction and increase oxidative stress. In vitro studies showed that LPS induces a shift of macrophages from oxidative phosphorylation to a glycolytic state, resulting in increased ROS production [182]. Another metabolite nicotinamide N-oxide, primarily produced by *Lactobacillus gasseri* and *Lactobacillus reuteri*, limited herpes simplex virus infection in the brain by restoring mitochondrial autophagy and preventing microglial hyperactivation in mice [183, 184]. Studies in untreated MS patients showed that propionic acid supplementation can reduce brain atrophy and restore mitochondrial function and morphology of Treg cells [185].

In patients with AD, mitophagy is dysregulated, and this dysregulation is associated with reduced levels of autophagy initiation proteins (p-TBK1 and p-ULK1) as well as decreased expression of mRNAs involved in mitochondria-associated autophagy [186, 187]. This causes a pathological buildup of dysfunctional mitochondria in neurons, which impairs synaptic plasticity and cognitive function. Clinical investigations further demonstrate a significant positive correlation between the severity of mitophagy impairment and disease progression across the spectrum from MCI to dementia [188].

Mitochondria are the primary site of ROS generation. ROS are produced as byproducts of oxidative phosphorylation [189, 190]. Superoxide dismutase 1 is the primary enzyme responsible for eliminating ROS. There is a strong correlation between AD and cerebral oxidative damage [191, 192]. It is worth noting that one of the main early pathological characteristics of AD is the increased ROS level caused by mitochondrial dysfunction. Aβ may directly promote ROS levels, and decreased SOD levels in patient neurons may also lead to ROS accumulation [193–195].

These findings reveal interactions between gut flora and mitochondria, which may be a key component of the Microbiota–Gut–Brain Axis. Further research is needed to develop precise diagnostics and personalized therapies targeting microbiota–mitochondrial communications.

#### Glucose metabolism

Positron emission tomography studies have revealed about 11% lower glucose utilization across the frontal, parietal, temporal, and cingulate lobes in patients with AD, compared to healthy controls. The decreased cerebral glucose metabolism is a hallmark of AD pathogenesis [196].

Gut microbiota may affect the course of AD by altering glucose metabolism. Administration of new formulations of *Lactobacillus* and *Bifidobacterium* in 3xTg-AD mice enhanced glucose uptake and reduced AD pathology in their brains, and improved their memory function [197]. Additionally, studies in mice showed that antibiotic-induced alterations in the gut microbiota, particularly reductions of *Firmicutes* and *Bacteroidetes*, can influence insulin sensitivity [198, 199]. Microbial metabolites such as SCFAs (like butyrate) may increase insulin sensitivity and reduce inflammation [200, 201], whereas trimethylamine-N-oxide, produced from the microbial metabolism of choline and carnitine, enhances glucose tolerance [202, 203].

One important risk factor for AD is type 2 diabetes mellitus (T2DM), and AD patients are more likely to develop T2DM. This reciprocal relationship raises the idea that AD is an insulin-resistant condition known as "type 3 diabetes" [204–206]. Insulin can cross the BBB through receptor-mediated transport to perform essential neuromodulatory actions in the CNS [207]. One of the main causes of neurological dysfunction is the disruption of insulin signaling in the brain.

Peripheral hyperinsulinemia impairs BBB permeability for insulin, which lowers the central insulin activity and, as a result, disturbs cerebral glucose homeostasis and neuronal function [208]. Insulin resistance can worsen AD pathology in several ways. First, insulin competitively prevents the removal of Aβ peptides by the insulin-degrading enzyme (IDE) in insulin-resistant individuals, leading to increased Aβ deposition [209–212]. Second, clinical research shows higher levels of total tau and phosphorylated tau in the CSF of patients with T2DM. Molecular research shows that the impaired insulin/insulin-like growth factor-1 signaling activates several tau kinases, such as GSK3β [213–216]. GSK3β is a key molecule in the Wnt/β-catenin signaling pathway. Wnt signaling regulates several developmental processes in the nervous system and has been implicated in the regulation of neurogenesis, dendritic morphogenesis and synaptic function [217–219]. Studies have shown that GSK-3β mediates the hyperphosphorylation of tau protein in AD patients [220, 221].

In summary, due to the extreme technical difficulty in accurately tracking the entry of intestinal flora metabolites into the circulatory system and across the BBB, further experiments are needed to validate their effects on glucose metabolism in brain cells.

### Gut microbiome and the autonomic nervous system

The enteric nervous system (ENS), often referred to as the 'second brain', is similar to the CNS in size, complexity, and neurotransmitters involved. Autonomic neurons of the vagus nerve make up the majority of the afferent neurons that transmit signals from the ENS to the CNS [222–224].

By altering gut hormones, neuropeptides, and cytokines, the ENS can either promote or inhibit the functioning of the HPA axis [225]. In C57BL/6 mice, both *Bacteroides* and *Escherichia* actively generate GABA (gamma-aminobutyric acid), which acts on the CNS and regulates the HPA axis via the ENS or vagus nerve [226, 227]. Furthermore, studies in germ-free mice using viral tracing and chemogenetic methods demonstrate that vagal sensory neurons and specific brainstem neuronal populations can respond to microbiota changes and regulate intestinal sympathetic nerve activity [228].

In animal models, celiac vagotomy failed to prevent the increase in blood and fecal LPS levels induced by *Escherichia fergusonii* or *Veillonella infantium*. However, it successfully prevented the associated cognitive impairment-like behaviors, as well as the increases in hippocampal TNF-α expression and NF-κB-positive cells. This dissociation indicates that these gut microbes affect the brain via an autonomic nervous system pathway that is independent of systemic LPS elevation [229].

APP is essential for normal gastrointestinal motility, immunity, and secretion. It is typically expressed in the ENS [230]. Progressive accumulation of Aβ has been found in the enteric neurons of transgenic mice expressing three mutant forms of APP. This Aβ accumulation is linked to decreased numbers of enteric neurons, impaired motor performance, and increased susceptibility to intestinal inflammation [231, 232]. The excessive accumulation of Aβ may even spread to the CNS through myenteric neurons, directly contributing to the pathogenesis of AD. However, the underlying mechanisms need to be investigated in the future [233–235].

## Oral microbiome and AD pathogenesis

Research on the gut-brain axis has revealed the close connections among gut microbiota, the nervous system, and the immune system, inspiring investigations into the interactions between other organs and the CNS. It is impossible to precisely characterize the oral microbiome in AD because the patients' oral hygiene practices change at the beginning of the disease. Despite this limitation, studies consistently report distinct microbial alterations in AD patients compared to controls. This reproducible pattern strongly suggests that oral dysbiosis is a significant contributing factor in AD etiology.

Many studies have suggested a close biphasic relationship between AD and oral disease, particularly periodontitis. Periodontitis is a chronic inflammatory disease mainly caused by dysbiosis of the subgingival microbial community, usually followed by increased levels of systemic inflammatory markers like prostaglandins, TNF-α, and IL-1 [11, 12, 236].

*Porphyromonas gingivalis* is a Gram-negative, anaerobic oral cavity bacterium that is one of the primary causes of chronic periodontitis. Recent studies suggest that *P. gingivalis* may increase the inflammatory load on the CNS, which could lead to cognitive deficits and be a risk factor for AD. In APP/PS1 mice and SD rats, injections of *P. gingivalis* LPS into the second molar resulted in periodontitis, which exacerbated Aβ accumulation, neuroinflammation, and microglial activation in the brain. In addition, inflammatory cytokines (TNF-α, IL-1β, IL-6, and IL-8) in the gingiva, peripheral blood, and hippocampus were significantly increased, resulting in worsening of cognitive impairment and anxiety-like behaviors [237, 238].

Another significant pathogen in chronic periodontitis is *Treponema denticola*, a gram-negative anaerobic bacterium. By infecting periodontal tissue or by modifying the immune system, *T. denticola* can directly or indirectly cause neurodegeneration. *T. denticola* given orally to C57BL/6 mice induced expression of IL-1β in the hippocampus, cortex, and midbrain, activation of astrocytes and microglia, and neuronal damage [239]. *T. denticola* was found to be able to enter the brain and directly impact neuronal cells, resulting in the accumulation of intracellular and extracellular Aβ_1-40_ and Aβ_1-42_ [240].

As a common Gram-negative anaerobic bacterium, *Fusobacterium nucleatum* has been shown to have interactions with microglia and frequently overgrows in periodontitis. In vivo studies in the 5 × FAD mouse model showed that *F. nucleatum* activates microglia, causing morphological changes, accelerating proliferation, and increasing the expression of TNF-α and IL-1β in microglia. Additionally, *F. nucleatum* increases Aβ buildup and tau protein phosphorylation, resulting in worsening of AD symptoms [241].

Furthermore, the onset of periodontitis in AD animals can disrupt intestinal homeostasis, leading to intestinal epithelial barrier breakdown and colonic inflammation [237]. Continuous gavage of periodontitis-associated salivary microbiota in APP/PS1 mice resulted in neuroinflammation, elevated Aβ buildup, and cognitive impairment. Consistently, intestinal proinflammatory responses, disruption of the intestinal barrier, gut microbial dysbiosis, and the exacerbation of systemic inflammation were observed [242]. These findings suggest that the periodontitis-associated microbiota may exacerbate cognitive impairment through the brain-gut axis.

Dental caries is another disorder associated with oral bacterial infections [11, 243]. Dental cavities may be connected with AD. In subgingival plaque samples from AD patients and healthy individuals, the moderate AD group had a higher relative abundance of the cariogenic dental pathogen *Lactobacillus* than the mild AD and healthy control groups [244]. A study assessing the oral health status of AD patients showed a strong correlation between dental caries and an elevated risk of AD [245, 246]. However, a dental caries–AD causality estimation showed that there was no association between dental caries and AD and that the genetic susceptibility to dental caries was not related to the onset age of AD [247]. This discrepancy might be explained by the focus of the cited studies: they assessed the clinical diagnosis of dental caries as a whole, rather than measuring the specific microbiological disturbances (e.g., pathogenic shifts) that drive the disease.

In conclusion, neurodegenerative disorders may result from changes in the composition and activity of oral microbiota. Further research is needed to elucidate how oral microbiota contributes to the development of AD, and whether preserving the integrity of the oral microbial community is an efficient strategy for AD prevention and treatment.

## The lung–brain microbial axis in AD

The lung**–**brain axis refers to the bidirectional signaling and regulatory network between the lungs and the CNS. The communication pathways between the lungs and brain include the neural, immune, endocrine, and circulatory systems. Known or potential mechanisms by which the CNS may be influenced by the lung microbiota include the direct translocation of microbes, as well as the effects of lung microbiota on systemic immunity, the nervous system, the HPA axis, and metabolism [16].

Compared with healthy controls, early-stage AD patients exhibit significant olfactory bulb and tract atrophy on MRI, accompanied by amyloid accumulation [248]. There is limited research exploring the impact of microbes on the development and progression of AD through the lung**–**brain axis. Existing research primarily focuses on identifying the mechanisms by which the novel coronavirus induces CNS symptoms and leads to long-term cognitive impairment.

Long COVID-19 is associated with long-term neurological symptoms, like brain fog, memory impairment, and speech and language difficulties [249, 250]. SARS-CoV-2 may cause cognitive impairment, neuronal loss, and neuroinflammation by the following mechanisms: direct attacks on the nervous system through olfactory pathways, retrograde transmission of the virus via the vagus nerve or through the BBB [251, 252]; activation of pulmonary immune cascades; disruption of various cellular organelles and biological processes in the cortex [253, 254]; infiltration of monocytes into the CNS, activation of microglia, vascular abnormalities, synaptic damage, and loss of astrocytes [255, 256]; induction of excessive inflammation by promoting NLRP3 inflammasome activation [257]; and over-phosphorylation of tau protein induced by the SARS-CoV-2 nucleocapsid protein [258].

In addition to the direct mechanisms of neural invasion and neuroinflammation as mentioned above, a range of host-specific factors also affect the impact of COVID-19 infection on the gut–brain axis and subsequent cognitive outcomes. The gut microbiome in older adults shows increased vulnerability to viral disruption, which may exacerbate neuroinflammatory responses [259]. Sex may be another potential factor; both animal and clinical studies have indicated that estrogen has notable anti-inflammatory effects [260, 261]. Additionally, decreased estrogen levels may lead to reduced intestinal microbial diversity [262]. Therefore, low estrogen levels may inhibit recovery from infection-induced dysbiosis and prolong the inflammatory response. Stress-vulnerable personality traits and lack of social support from, e.g., caregivers, may exacerbate systemic inflammation and disrupt the gut–brain axis, potentially worsening the memory deficits in long COVID patients [263]. Caregivers of AD patients also frequently experienced anxiety and depressive symptoms during the pandemic [264, 265]. These mental symptoms represent a potential pathway that could disrupt the microbiota–gut–brain axis through activation of the HPA axis, barrier dysfunction, and persistent inflammation—a cascade known to exacerbate neuroinflammation and cognitive decline [266, 267]. However, direct evidence linking caregiver distress to such neurological outcomes in themselves is currently lacking [268].

In mice, *Bordetella pertussis* infection leads to Aβ deposition and inflammation [269]. In another study, old mice show more severe changes in gene expression related to cognitive dysfunction and memory impairment compared to young mice after non-neurotrophic Influenza A virus infection [270]. Patients recovering from pneumonia often exhibit long-term cognitive impairment comparable to those seen in AD patients. Lung endothelial cells exposed to *Pseudomonas aeruginosa* bacterial infection produce and release cytotoxic tau species in the cell media, which induces neuronal tau aggregation [271].

**Fig. 3:**
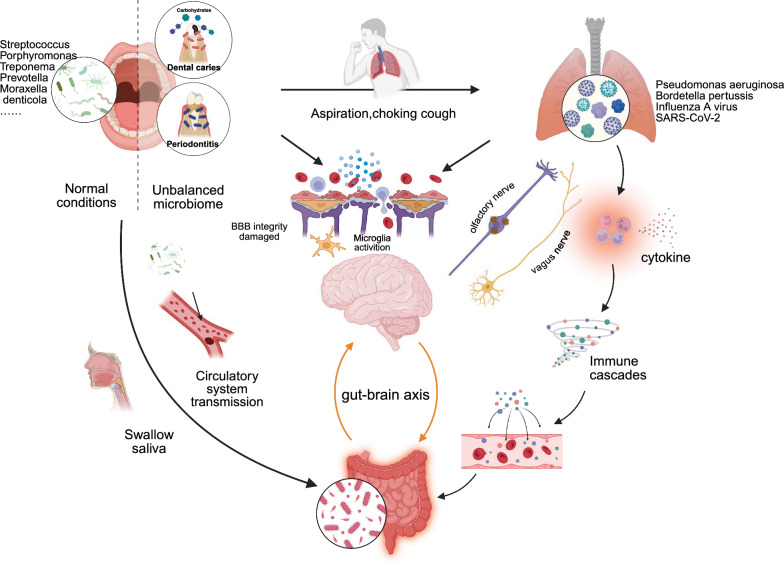
The “Multi-Axis Co-Regulation” concept in Alzheimer’s disease. The figure illustrates how the microbiota of the gut, mouth, and lungs interact through immune, metabolic, and neural pathways to  jointly participate in the pathogenesis of AD

Although the individual roles of the gut, oral, and pulmonary microbiota in AD pathogenesis are increasingly recognized, the existing research often examined them in separation. We hereby propose a more integrative “Multi-Axis Co-Regulation” model (Fig. 3), in which the distinct microbial communities do not act independently but rather form a dynamic, interconnected network that collectively modulates AD progression through coordinated mechanisms.

We hypothesize that the “Multi-Axis Co-Regulation” model operates via several interrelated pathways: (1) microbial translocation. Microbiota can colonize from one site to another via blood, saliva, etc. For instance, studies indicate that oral bacteria can colonize the gut and trigger inflammatory responses [62, 272]; (2) inflammatory mechanisms. Chronic, mild inflammation at one site (e.g., periodontitis) can activate immune cells and alter their response to stimuli from another specific site (e.g., gut microbiota dysbiosis), creating a synergistic pro-inflammatory environment that further intensifies neuroinflammation; (3) metabolite integration. Microbial metabolites (such as SCFAs from the gut, LPS from the oral bacteria, and circulating inflammatory mediators from the lungs, etc.) can be transmitted through the lymphatic and circulatory systems [273, 274], forming a comprehensive metabolic signal that directly affects neuronal health and function.

## Extra-intestinal microbiota in AD

Apart from the microbiota of the lungs, mouth, and intestines, dysbiosis in other bodily regions may also directly or indirectly contribute to the pathophysiology of AD.

Nasal cavity is one of the most important routes for CNS infection. The microbial flora in the nasal cavity can bypass the BBB and enter the brain via the olfactory system, or through the circulatory system when the BBB is damaged [16]. Mice that were intranasally infected with *Chlamydia pneumoniae* developed amyloid plaques in their brains, resembling those seen in AD patients [275]. Additionally, *C. pneumoniae* has been found in astrocytes and microglia in AD patient brains [276], which may impair the functions of glial cells, hence accelerating the course of AD. Furthermore, diphtheria toxin produced by *Corynebacterium diphtheriae* may enter the entorhinal cortex and spread to the hippocampus, contributing to AD development, and vaccination provides protection against diphtheria toxin [277]. After entering the brain, some nasal viruses, such as Herpes simplex virus (HSV), can cause microglial release of pro-inflammatory cytokines, which exacerbate neuroinflammation [278]. Additionally, the HSV-1 glycoprotein B fragment is homologous to several Aβ peptides, which may form beta-pleated sheets, self-assemble into fibrils that are ultrastructurally indistinguishable from Aβ, thereby accelerating the synthesis of Aβ [279].

Skin is the biggest organ of the human body, and is essential for protecting the body from infection. Although the underlying mechanisms remain incompletely understood, Aβ and tau proteins have been detected in skin biopsies of patients with AD. Moreover, certain dermatological conditions exhibit complex associations with AD. For example, melanoma cells have been shown to secrete Aβ, which may promote tumor growth, while patients with psoriasis demonstrate an increased risk of developing AD—a link potentially mediated by inflammatory factors such as TNF-α [280]. Microbial infections of the skin have also been proposed as a risk factor for AD. In animal models, *Malassezia* infections associated with skin damage facilitate bacterial infiltration into the brain and induce microglial phagocytic dysfunction, along with upregulation of IL-17, IL-23, and TLR4 expression [281]. Thus, it may be concluded that *Malassezia* infection may worsen neuroinflammation and enhance Aβ buildup. As previously stated, HSV-1 is closely linked to AD. Varicella-zoster virus infection of cells quiescently infected with HSV-1 causes reactivation of HSV-1 and accumulation of Aβ and p-tau [282].

## Microbial signatures for AD diagnosis

In recent years, the potential of microbiota in AD diagnosis has received extensive attention. Research on gut and oral microbiota, in particular, has provided new diagnostic insights (Table 3).

**Table 3:** Summary of research on the application of microbial biomarkers in the diagnosis of AD

Reference	Year	Participants	Analysis approach	Main finding
[333]	2017	AD (*n* = 25); Control (*n* = 25)	16S rRNA amplicon sequencing	AD patients showed a decrease in *Firmicutes* and *Bifidobacterium*, and an increase in *Bacteroidetes*
[334]	2017	CI with Aβ (*n* = 40); CI without Aβ (*n* = 33); control (*n* = 10)	Microbial DNA qPCR Assay Kit	Compared to healthy controls and amyloid-negative individuals, amyloid-positive individuals had lower levels of anti-inflammatory *Eubacterium rectale* and higher levels of inflammatory *Escherichia/Shigella*
[335]	2017	Cognitively intact group (*n* = 25); CI (*n* = 18)	16S rRNA amplicon sequencing	The cognitively intact group had lower proportions of *Bacteroidetes* and *Proteobacteria*, and higher proportions of *Firmicutes* and *Verrucomicrobia*
[336]	2018	AD (*n* = 43); control (*n* = 43)	16S rRNA amplicon sequencing	AD patients showed a decrease in *Bacteroidetes* and an increase in *Actinobacteria*
[384]	2019	AD (*n* = 33); aMCI (*n* = 32); control (*n* = 32)	16S rRNA amplicon sequencing	Compared to the control group, AD patients showed a decrease in *Firmicutes* and an increase in *Proteobacteria*, with a gradual increase in the enrichment of *Gammaproteobacteria*, *Enterobacterales*, and *Enterobacteriaceae* from the control group to aMCI and AD
[385]	2019	Demented (*n* = 34); control (*n* = 94)	16S rRNA amplicon sequencing	Dementia patients showed a decrease in *Bacteroidetes* and a lower *Firmicutes/Bacteroidetes* ratio
[386]	2019	AD (*n* = 30); aMCI (*n* = 30); control (*n* = 30)	16S rRNA amplicon sequencing	Eleven bacterial genera were identified that differed between AD and healthy controls, but no significant differences were found between AD and MCI. A diagnostic model based on fecal samples correctly identified 93% (28/30) of MCI patients
[387]	2019	MCI (*n* = 61); control (*n* = 21)	Structural MRI 16S rRNA amplicon sequencing	MCI patients with higher levels of *Bacteroidetes* were more likely to exhibit significant cortical and hippocampal atrophy
[388]	2020	AD (*n* = 88); control (*n* = 65)	ITS2 rRNA sequencing	*Candida tropicalis* and *Schizophyllum* commune were enriched in AD patients, while *Rhodotorula mucilaginosa* was significantly reduced
[389]	2020	AD (*n* = 100); control (*n* = 71)	16S rRNA amplicon sequencing	AD patients showed a decrease in *Faecalibacterium* and an increase in *Bifidobacterium*
[390]	2021	MCI (*n* = 75); Control (*n* = 52)		MCI participants had lower abundances of *Faecalibacterium*, unclassified *Ruminococcaceae*, and *Alistipes*, while *Proteobacteria* and *Gammaproteobacteria* increased. The combination of diet quality, gut microbiota, and serum miRNAs effectively distinguished MCI (AUC = 0.91)
[391]	2021	CI (*n* = 14); SCD (*n* = 53); control (*n* = 38)	16S rRNA amplicon sequencing	The abundance of anti-inflammatory *Faecalibacterium* was significantly reduced in SCD
[392]	2021	aMCI (*n* = 20); control (*n* = 22)	16S rRNA amplicon sequencing	*Bacteroidetes* were higher in aMCI compared to the control group, while *Firmicutes* and *Proteobacteria* showed no significant differences
[393]	2021	AD (*n* = 18); MCI (*n* = 20); control (*n* = 18)	16S rRNA amplicon sequencing	AD and MCI patients showed a decrease in *Bacteroidetes*, *Lachnospira*, and *Ruminiclostridium 9*, and an increase in *Prevotella*
[394]	2021	MCI (*n* = 22); control (*n* = 26)	16S rRNA amplicon sequencing	The relative abundance of *Bacteroidetes* was lower in MCI, while the relative abundance of *Fusobacteria* was significantly higher in MCI
[395]	2021	AD (*n* = 60); control (*n* = 32)	16S rRNA amplicon sequencing	AD patients showed enrichment of *Bifidobacterium*, *Sphingomonas*, *Lactobacillus*, and *Blautia*, while *Odoribacter*, *Anaerotruncus*, and *Papillibacter* were reduced
[396]	2022	AD (*n* = 11); MCI (*n* = 11); CN + (*n* = 32); CN − (*n* = 34)	16S rRNA amplicon sequencing	Compared to CN − patients, CN + patients showed a decrease in *Firmicutes* and *δ-Proteobacteria*, and an increase in *Bacteroidetes*. The combination of plasma Aβ and changes in gut microbiota could be used to identify CN +
[397]	2022	AD (*n* = 45); control (*n* = 54)	16S rRNA amplicon sequencing	The gut microbiota of AD patients showed no significant differences compared to the control group, while the oral microbiota showed significant differences
[398]	2023	CI (*n* = 30); SCD (*n* = 62); control (*n* = 35)	16S rRNA amplicon sequencing	With improved cognitive ability, the abundance of *Lachnospiraceae*, *Fusicatenibacter, Lachnospiracea_incertae_sedis*, and *Anaerobutyricum* decreased, while *Rikenellaceae*, *Alistipes*, and *Odoribacteraceae* were enriched in the CI group
[399]	2023	Preclinical AD (*n* = 49); control (*n* = 115)	Metagenomic sequencing	*Dorea*, *Oscillibacter 57_20*, *Faecalibacterium prausnitzii*, *Coprococcus*, and *Anaerostipes hadruti* were most associated with preclinical AD status. Changes in the gut microbiome were related to Aβ and tau
[400]	2024	Older adults (*n* = 605)	16S ribosomal RNA gene sequencing	Oral microbiota composition is associated with executive function and subjective memory changes in middle-aged and older adults. Oral dysbiosis is a potential biomarker or therapeutic target for cognitive decline
[401]	2024	AD (*n* = 32); aMCI (*n* = 32); Control (*n* = 32)	16S rRNA gene sequencing, LC–MS/MS analysis	Periodontal microbial dysbiosis and metabolic disturbances may be involved in the onset and progression of AD and could serve as potential biomarkers
[402]	2024	AD (*n* = 84); MCI (*n* = 84); At-risk individuals (APOE4 carriers; *n* = 17); Control (*n* = 50)	16S rRNA analyses	AD patients showed changes in the abundance of *Bacteroides*, *Ruminococcus*, *Sutterella*, and *Porphyromonadaceae*. Additionally, the oral microbiota diversity increased in AD and high-risk individuals, with enrichment of Gram-negative pro-inflammatory bacteria (*Haemophilus*, *Neisseria*, *Actinobacillus*, and *Porphyromonas*), whose abundance was associated with cerebrospinal fluid biomarkers
[403]	2024	CI (*n* = 74); NCI (*n* = 131)	The linear discriminant analysis effect size method and machine learning approaches	CI group showed lower α-diversity and a decrease in gut microbial interaction network density, with *Megamonas*, *Blautia, Pseudomonas*, *Dysgonomonas*, and *Veillonella* being key biomarkers for CI

Aβ, Amyloid-beta; AD, Alzheimer's disease; APOE4, Apolipoprotein E4; AUC, Area Under the Curve; CI, Cognitive Impairment; CN + , Aβ-positive Cognitively Normal; CN − , Aβ-negative Cognitively Normal; ITS2, Internal Transcribed Spacer 2; LC–MS/MS, Liquid Chromatography-Tandem Mass Spectrometry; MCI, Mild Cognitive Impairment; aMCI, Amnestic Mild Cognitive Impairment; miRNAs, microRNAs; MRI, Magnetic Resonance Imaging; NCI, No Cognitive Impairment; Preclinical AD, Preclinical Alzheimer's disease; qPCR, Quantitative Polymerase Chain Reaction; SCD, Subjective Cognitive Decline; 16S rRNA, 16S ribosomal RNA gene

Current research trends focus on combining microbiota with brain biomarkers, such as changes in Aβ and tau proteins, to improve diagnostic accuracy through integrated analysis. However, differences in research cohorts and sample types may lead to varied results of microbiome changes across studies [283–285]. Therefore, rigorous quality control and adjustment for covariates such as diet are needed in future studies. Advanced analytical approaches, including machine learning, could be employed to identify highly associated microbial features.

Another research direction is to identify early biomarkers of AD based on the metabolite profile of the microbiome. For example, dysbiosis of the periodontal microbiota and metabolic disturbances have been considered potential contributors to the onset and progression of AD, highlighting the value of detecting microbial metabolites through non-invasive methods [286]. Specific microbial metabolites, such as SCFAs and LPS, may be closely associated with the pathological processes of AD. A 2019 study found that LPS and fragments of Gram-negative *Escherichia coli* co-localized with amyloid plaques in the post-mortem brain tissues of AD patients [287]. In a 2021 study, combined use of gut microbiota composition, serum miRNAs, and dietary quality scores greatly improved the reproducibility and consistency of diagnosis of MCI from healthy individuals [288].

Nevertheless, these diagnostic methods have certain limitations. Firstly, the diversity of the microbiome and individual differences are significant, especially since dietary and lifestyle choices can significantly alter the gut microbiota. It is necessary to further explore whether there are specific microbial changes in AD patients [289]. For instance, studies have shown that a high fecal abundance of *Prevotellaceae* may serve as a potential biomarker for excluding PD [290]. In HD, the fecal abundance of *Eubacterium hallii* exhibits a significant negative correlation with motor symptom severity, estimated time to onset, and cognitive performance [291]. Moreover, most studies rely on cross-sectional analyses and lack sufficient longitudinal data to validate the causal relationship between microbiome changes and the pathogenesis of AD. Therefore, future research needs to further increase sample sizes, consider the effects of age and gender, and optimize data integration methods.

## Microbiota-targeting therapies for AD

**Fig. 4:**
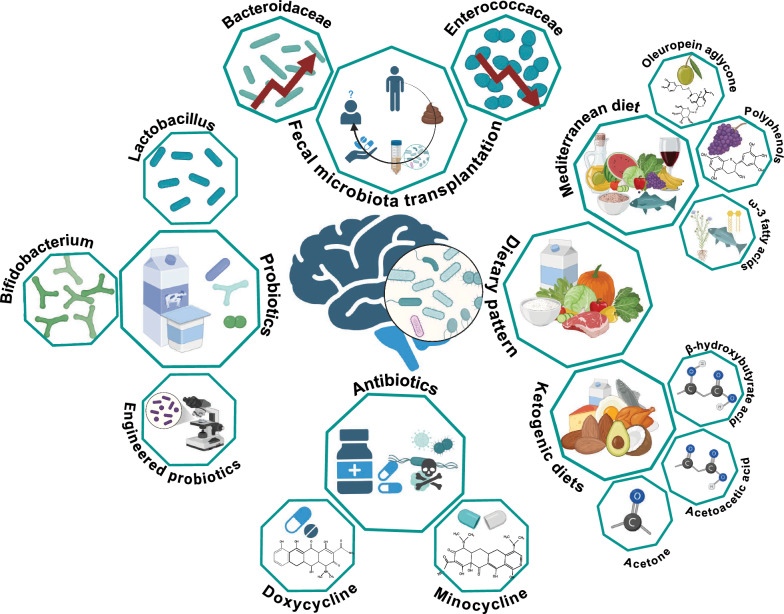
Microbiota in Alzheimer's disease treatment. Microbiota-targeting interventions, including FMT, probiotics, dietary modulation, and antibiotic treatments, are emerging as promising strategies for mitigating AD

Dysbiosis of the microbiome may impact host immunity and metabolism, thereby influencing the onset and progression of AD. Based on these effects, microbiome intervention strategies to restore microbiota balance, including probiotics, FMT, and dietary regulation (such as the Mediterranean diet), have the potential to slow down or prevent the onset and development of AD (Fig. 4).

### Probiotics

Probiotics is a low-risk, safe therapeutic option that has shown encouraging promise in the prevention and treatment of AD. Specific probiotics can stabilize the intestinal barrier, reduce intestinal and BBB permeability, and ameliorate the gingival epithelial barrier damage [292–295]. Orally administering *L. plantarum* PS128 prevents activation of astrocytes and microglia in the mouse striatum [296]. Intragastrical *Clostridium butyricum* therapy prevented microglial activation, Aβ deposition, and cognitive impairment in APP/PS1 mice [297]. A randomized controlled study in healthy adults demonstrated that fermented milk containing *L. rhamnosus* SD11 enhances oral health by increasing salivary *Lactobacilli* and decreasing *S. mutans* and other bacterial populations [298].

The application of probiotics still faces obstacles due to the individual differences, a lack of knowledge about the underlying mechanisms, etc. The dosage, the percentage of strains, and the severity of the illness can influence the efficacy of probiotics. Different strains have varying effects on the pathological changes of AD. *L. plantarum* ATCC 8014, for example, decreased Aβ plaques in mice but only marginally impacted hippocampal neuronal plasticity [299]. In contrast, *L. plantarum* PS128 did not significantly alter tau protein level or Aβ deposition in mice, but it decreased microglial activation [300]. *Bifidobacterium breve* A1 had no effect on tau phosphorylation, but it decreased the amount of Aβ produced in mouse hippocampus [301]. Furthermore, clinical research has demonstrated that the sensitivity to probiotic treatment varies among individuals with different disease severities. Probiotics are not very effective for patients with severe AD, but they are more effective for preclinical-stage AD [302].

Engineered probiotics have emerged as a novel drug delivery system, overcoming the limitations of traditional probiotics including a low treatment success rate and limited specificity. In animal studies, oral probiotic *Escherichia coli* Nissle 1917, which was genetically engineered to overexpress catalase and superoxide dismutase for the treatment of intestinal inflammation, effectively reduced intestinal inflammation, repaired the intestinal epithelial barrier, and modulated intestinal microbiota [303]. Engineered probiotics is a promising direction with multi-target efficacy and low toxicity. Nevertheless, further research is still required to determine the biosafety issues.

### FMT

FMT is a method that uses a variety of procedures to prepare the feces of healthy donors and transplant them into the gastrointestinal tract of patients. This directly alters the recipient's gut microbiota, restores microbial balance, and provides therapeutic benefits [304, 305].

Preliminary studies suggest that FMT may delay the progression of AD by modifying gut microbiota, altering microbial metabolites, lowering inflammation, improving Aβ metabolism, and enhancing cognitive function [55]. Antibiotic-treated 5 × FAD mice fed with cecal suspensions from normal mice exhibited an increase in representatives of *Enterobacteriaceae* and *Lactobacillaceae* and a decrease in *Firmicutes* in the feces [306]. Orally administering fresh fecal solutions from C57BL/6 male mice in APP/PS1 male mice restored the gut microbiota dysbiosis and significantly increased the SCFA butyrate level in AD mice. This also decreased Aβ deposition in the brain, tau phosphorylation, and levels of Aβ_40_, Aβ_42_, COX-2, and CD11b, and ameliorated cognitive deficits [307–309]. In five AD patients who received FMT, an increase of *Bacteroidaceae* and a decrease of *Enterococcaceae* in their gut microbiota were observed, along with improved cognitive function [310]. In the R6/1 HD mouse model, FMT appeared to have limited overall efficacy and exhibited sex-specific effects [311], suggesting a sex-specific effect of FMT for AD treatment.

Although FMT has shown potential benefits in the prevention and treatment of AD, its clinical application still faces numerous challenges. The primary issue is the lack of clinical trials. Given the possibility of infection or rejection in AD patients with compromised immune system, the safety of FMT cannot be overlooked. Additionally, the individual differences in gut microbiota make it difficult to predict the therapeutic effects of FMT. To address these issues, researchers need to further optimize the donor selection criteria, improve treatment protocols, and identify specific beneficial microbiota to enhance the stability and safety of the transplant. FMT can also be combined with other intervention strategies, such as prebiotics, dietary regulation, and anti-inflammatory therapies, to improve the treatment effect.

### Dietary pattern

Diet is a key factor in maintaining health and protecting against oxidative stress and chronic inflammation, thereby preventing chronic degenerative diseases [312]. An "animal-based diet" (primarily consisting of meat, eggs, and cheese) can enhance the β-diversity of the gut microbiota, while a "plant-based diet" (rich in grains, legumes, fruits, and vegetables) can elevate levels of SCFAs. A high-sugar diet (rich in glucose, fructose, and sucrose) is closely associated with increased abundance of *Bifidobacterium* and decreased abundance of *Bacteroides*. Meanwhile, undigested carbohydrates can function as prebiotics, positively influencing gastrointestinal function by promoting the growth of probiotic bacteria [313]. Current research mainly focuses on ketogenic diet and the Mediterranean diet.

The ketogenic diet is a high-fat, adequate-protein, and very-low-carbohydrate diet that induces ketosis. In this state, the body uses fat as a main energy source and produces metabolic byproducts known as ketone bodies (KET), which provide energy to the brain and other organs [314]. Brain cells in AD patients often cannot effectively utilize glucose, whereas KET can serve as an alternative energy source to support the brain [315]. Basic research has found that the ketogenic diet exerts neuroprotective effects by inhibiting neuroinflammation and ROS production, decreasing Aβ deposition and microglial activation, protecting dopaminergic neurons, inhibiting tau hyperphosphorylation, stimulating mitochondrial biogenesis, enhancing gut microbiome diversity, restoring histone acetylation, and promoting neuronal repair [316].

The Mediterranean diet is a typical high-fiber diet, which represents the typical dietary pattern of populations along the Mediterranean coast. This diet promotes natural, fresh, and unprocessed foods. Moderate wine drinking is also encouraged [317, 318]. The Mediterranean diet offers potential in the prevention and treatment of AD due to its rich content of antioxidants, healthy fats, and anti-inflammatory components. A 2021 clinical study concluded that the Mediterranean diet could protect against memory decline and atrophy of the middle temporal lobe [319]. AD-related research mainly focuses on the active substances in the food, such as oleuropein aglycone in extra virgin olive oil, polyphenols in red wine, and omega-3 fatty acids found in deep-sea fish and flaxseeds.

The two dietary patterns have been merged to develop the modified Mediterranean-ketogenic diet (MkD). This diet changes the gut microbiome and microbial metabolites, particularly promoting the growth of lactic acid bacteria. Additionally, in a transgenic AD mouse model, the MkD elevated the levels of specific microbiome- and diet-derived metabolites in the bloodstream, upregulated neuroprotective receptors, and modulated neuroinflammation-related pathways in the hippocampus [320].

In addition to the neuroprotective effects of individual active components, the combined intake of different types of foods may exert more comprehensive effects on the nervous system through synergistic mechanisms. For instance, long-term consumption of a Western diet—characterized by high fat, high carbohydrates, and high calories—downregulates the expression of intestinal antimicrobial peptides (AMPs) [321]. AMPs play a key regulatory role in maintaining immune tolerance of the gut microbiota and preventing pathogen colonization [322]. Direct evidence linking AMPs to AD remains limited, and current understanding is largely based on indirect mechanistic inferences—for instance, that they may influence AD progression by maintaining gut microbial balance and mitigating systemic inflammation. Interestingly, an animal study revealed an opposing mechanism: under conditions of infection or inflammation, upregulation of the human AMP LL-37 can cause hyperactivation of glial cells, leading to neuroinflammation and excitotoxicity, ultimately promoting neuronal death and accelerating AD pathogenesis [323].

Oral microbiota composition can also be influenced by dietary choices. Significant antibacterial qualities can be found in foods like garlic, olive oil, curcumin, and cinnamon. Regular ingestion of these foods may alter the composition and structure of the oral microbiota [11].

However, the efficacy of dietary patterns may be influenced by hormonal profiles, immune responses, long-term adherence, and significant individual variations [324, 325]. Future research may also explore the synergistic effects of dietary patterns with lifestyle factors like cognitive training and exercise, leading to a comprehensive strategy for AD prevention and treatment.

### Antibiotic-based therapeutic approaches

Broad-spectrum antibiotics can significantly impact the composition of the gut microbiota, reduce its biodiversity, and delay bacterial colonization for a long period after administration [326]. Therefore, antibiotics may have potential applications in the prevention and treatment of AD.

Research on the use of antibiotics to treat neurological diseases is still in progress. Doxycycline and minocycline, in particular, have demonstrated anti-inflammatory effects by inhibiting the activation of microglia and macrophages. Their efficacy as anti-inflammatory agents has been confirmed in experimentally induced neurodegenerative disease models both in vitro and in vivo [327, 328]. For example, minocycline ameliorated memory dysfunction by restoring acetylcholinesterase and ChRM1 levels, balancing oxidants/antioxidants, and inhibiting inflammatory responses in an AD mouse model [329]. Similar outcomes have been shown for doxycycline. Doxycycline inhibits amyloidosis by binding tightly to hydrophobic amino acids that are exposed on the Aβ42 amyloid fibrils, partially destabilizing the fibril structure [330]. However, research on the use of antibiotics for treating AD is still limited, especially with a lack of human-based studies.

However, widespread use of antibiotics may lead to gut microbiota dysbiosis and even the emergence of drug-resistant strains, potentially causing adverse effects. To address these potential negative impacts, researchers could develop targeted antibiotics for specific bacterial strains in future studies to precisely modulate the microbiota.

## Conclusion and outlook of precision microbiome strategies for AD

In this review, we first focus on research progresses in the brain-gut axis, and further explore the potential impact of oral, pulmonary, nasal, and skin microbiomes on AD. These microbial communities are not isolated but rather interconnected through natural anatomical pathways such as the respiratory and digestive tracts. For example, oral microbes can migrate to the lungs via aspiration, while the lung microbiota can regulate systemic inflammation levels to influence the gut environment. Based on these observations, we propose the “Multi-Axis Co-Regulation” concept, suggesting that microbiota from different body sites may jointly modulate the pathogenesis of AD. This concept emphasizes the importance of investigating microbial crosstalk across body sites in future studies and accounting for the potential confounding effects of cross-site microbial interactions in experimental designs.

Currently, there are several key challenges in elucidating the association between microbiota and AD. First, the underlying mechanisms remain poorly understood. For example, the specific pathways through which microbial metabolites enter the bloodstream and cross the BBB remain unclear, which limits clinical translation. Second, the gut environment of humans exhibits significant differences from that of mice, in compositions and functions of the microbiome. Therefore, results from mouse studies must be interpreted with caution when extrapolated to humans. Third, the widespread use of antibiotics to eradicate microbial populations may disrupt the internal equilibrium of microbial communities. This approach overlooks synergistic interactions among different microorganisms, making it difficult to accurately reflect the holistic impact of the microbiome on disease. To address these issues, future research should take in account multi-site microbial interactions and synergistic effects among different microbial communities during experimental design. Concurrently, experimental models more closely resembling humans should be established.

While this review focuses on the role of the microbiota in AD, a central question remains: to what extent are these findings specific to AD? Although dysregulation of the gut–brain axis represents a common feature across neurodegenerative disorders such as PD and HD, the specificity of AD may stem from its distinct pathological core. First, the microbiome or its metabolites may directly or indirectly modulate the production and clearance of Aβ or influence the phosphorylation and aggregation of tau. Second, AD-associated neuroinflammatory patterns, particularly microglial responses to Aβ plaques, may be uniquely regulated by gut-derived signals. Therefore, future research should not only employ longitudinal cohorts to establish the temporal relationship between microbial changes and AD progression, but also conduct cross-disease comparisons and mechanistic experiments to identify the specific microbial taxa and functional pathways that drive AD pathology.

## Data Availability

Not applicable.

## Abbreviations

Aβ: β-Amyloid
AD: Alzheimer's disease
BBB: Blood–brain barrier
CA: Cholic acid
COX-2: Cyclooxygenase-2
DCA: Deoxycholic acid
FMT: Fecal microbiota transplantation
FXR: Farnesoid X receptor
HD: Huntington’s disease
HDACs: Histone deacetylases
HPA: Hypothalamic–pituitary–adrenal
HSV: Herpes simplex virus
IAAld: Indole-3-acetaldehyde
IAA: Indole-3-acetic acid
IDE: Insulin-degrading enzyme
IPA: Indole-3-propionic acid
IPyA: Indole-3-pyruvic acid
KET: Ketone bodies
LCA: Lithocholic acid
LPS: Lipopolysaccharides
MkD: Mediterranean-ketogenic diet
Nrf2: Nuclear factor erythroid 2-related factor 2
PD: Parkinson’s disease
ROS: Reactive oxygen species
SCFAs: Short-chain fatty acids
T2DM: Type 2 diabetes mellitus
TCA: Taurocholic acid
Tregs: Regulatory T cells
TUDCA: Tauroursodeoxycholic acid
